# Extract from a mutant *Rhodobacter sphaeroides* as an enriched carotenoid source

**DOI:** 10.3402/fnr.v60.29580

**Published:** 2016-03-31

**Authors:** Chih-Chiang Wang, Shangwu Ding, Kuo-Hsun Chiu, Wen-Sheng Liu, Tai-Jung Lin, Zhi-Hong Wen

**Affiliations:** 1 Department of Marine Biotechnology and Resources, National Sun Yat-sen University, Kaohsiung, Taiwan; 2 Department of Medicine, Kaohsiung Armed Forces General Hospital, Kaohsiung, Taiwan; 3 Department of Chemistry, National Sun Yat-sen University, Kaohsiung, Taiwan; 4 Department and Graduate Institute of Aquaculture, National Kaohsiung Marine University, Kaohsiung, Taiwan; 5 Asia-Pacific Biotech Developing, Inc., Kaohsiung, Taiwan; 6 Department of Pharmacy and Graduate Institute of Pharmaceutical Technology, Tajen University, Pingtun County, Taiwan

**Keywords:** anti-oxidative, *Rhodobacter sphaeroides*, Lycogen^TM^, phototrophic bacteria, carotenoid, methoxyneurosporene

## Abstract

**Background:**

The extract Lycogen™ from the phototrophic bacterium *Rhodobacter sphaeroides* (WL-APD911) has attracted significant attention because of its promising potential as a bioactive mixture, attributed in part to its anti-inflammatory properties and anti-oxidative activity.

**Objective:**

This study aims to investigate the components of Lycogen™ and its anti-inflammatory properties and anti-oxidative activity.

**Design and results:**

The mutant strain *R. sphaeroides* (WL-APD911) whose carotenoid 1,2-hydratase gene has been altered by chemical mutagenesis was used for the production of a new carotenoid. The strain was grown at 30°C on Luria–Bertani (LB) agar plates. After a 4-day culture period, the mutant strain displayed a 3.5-fold increase in carotenoid content, relative to the wild type. In the DPPH test, Lycogen™ showed more potent anti-oxidative activity than lycopene from the wild-type strain. Primary skin irritation test with hamsters showed no irritation response in hamster skins after 30 days of treatment with 0.2% Lycogen™. Chemical investigations of Lycogen™ using nuclear magnetic resonance (NMR) ^1^H, ^13^C, and COSY/DQCOSY spectra have identified spheroidenone and methoxyneurosporene. Quantitative analysis of these identified compounds based on spectral intensities indicates that spheroidenone and methoxyneurosporene are major components (approximately 1:1); very small quantities of other derivatives are also present in the sample.

**Conclusions:**

In this study, we identified the major carotenoid compounds contained in Lycogen™, including spheroidenone and methoxyneurosporene by high-resolution NMR spectroscopy analysis. The carotenoid content of this mutant strain of *R. sphaeroides* was 3.5-fold higher than that in normal strain. Furthermore, Lycogen™ from the mutant strain is more potent than lycopene from the wild-type strain and does not cause irritation in hamster skins. These findings suggest that this mutant strain has the potential to be used as an enriched carotenoid source.

Carotenoids are an important and diverse class of widely distributed natural pigments produced by numerous microorganisms and plants. Carotenoids play a major role in the protection of plants against photooxidative processes (1–3). They have been used commercially as animal feed supplements, food colorants, and cosmetic additives (4). Carotenoids are efficient free-radical scavengers, exhibiting antioxidant activity; in addition, they enhance the vertebrate immune system. In the human organism, carotenoids are part of the antioxidant defense system. According to their structures, most carotenoids exhibit absorption maxima at around 450 nm. Filtering of blue light has been proposed as a mechanism for protecting the macula lutea against photooxidative damage (5–7). Carotenoids are also used for medical and biotechnological purposes and as potent antimicrobial agents (8, 9). Humans and animals are largely incapable of synthesizing carotenoids and, therefore, must obtain them from their diet.

Microbial carotenoids have many advantages over carotenoids from plants (3, 10, 11). One such advantage is related to fermentation, which is an inherently faster and more efficient process compared with other production methods. The other enduring strength of microbes is their relatively large and easily manipulated chromosomes. In addition, microorganisms produce carotenoids in different hues that are independent of weather conditions, and grow on inexpensive substrates (12).

In phototrophic bacteria, carotenoids are essential components of the photosynthetic process, providing a mechanism for photoprotection against autooxidation. They also participate in the energy-transfer process. Phototrophic bacteria can accumulate several different forms of carotenoids (13), which are essential compounds contained in photosynthetic intercytoplasmic membranes of phototrophic bacteria (14–16).

Carotenoid 1,2-hydratase (crtC), member of the hydro-lyases group, appears in the biosynthetic pathway of different acyclic carotenoids in photosynthetic bacteria. crtC introduces a tertiary hydroxy group into a carotenoid molecule by the addition of water to the carbon–carbon double bond at the C-1 position. Several crtC have been identified in photosynthetic bacteria. The crtC was found to be a membrane-bound enzyme with a molecular weight of 44 kDa (17). *Rhodobacter sphaeroides* is one kind of photosynthetic bacterium, containing crtC gene and producing carotenoids. Lycogen™ is the methanol extract of a mutant strain, *R. sphaeroides* (WL-APD911) whose crtC gene has been altered by random chemical mutagenesis, leading to the production of a new carotenoid (18).

Lycogen™ has attracted significant attention because of its promising biotechnological potential as demonstrated in our previous studies (18–20). For example, Wu and Liu reported that *R. sphaeroides* (WL-APD911) grew well in LB medium, and the extract Lycogen™ had the ability to inhibit NO production and iNOS expression in LPS-induced RAW 264.7 cells. In addition, Liu et al. used a novel strategy utilizing Lycogen™ as a potent anti-inflammatory agent to treat dextran-sodium sulfate (DSS)-induced colitis (21). The oral administration of Lycogen™ has been shown to reduce the expression of pro-inflammatory cytokines in mice. In addition, Lycogen™ has been shown to ameliorate the bacterial flora in the colon induced by DSS. Furthermore, Lycogen™ showed more potent antioxidative effects and less cytotoxicity, compared with lycopene (20). Lycogen™ has been proven to improve glucose homeostasis in streptozotocin-induced diabetic mice (20). Because of its promising biotechnological potential, the quantitative information about its composition is essential. In this work, we used high-resolution nuclear magnetic resonance (NMR) spectroscopy to determine the major components of Lycogen™. We also used a DPPH test to compare the antioxidative activity of Lycogen™ and lycopene from the wild-type strain. Moreover, we carried out a skin irritation test with hamsters to check the biocompatibility of Lycogen™.

## Materials and methods

**Fig. 1:**
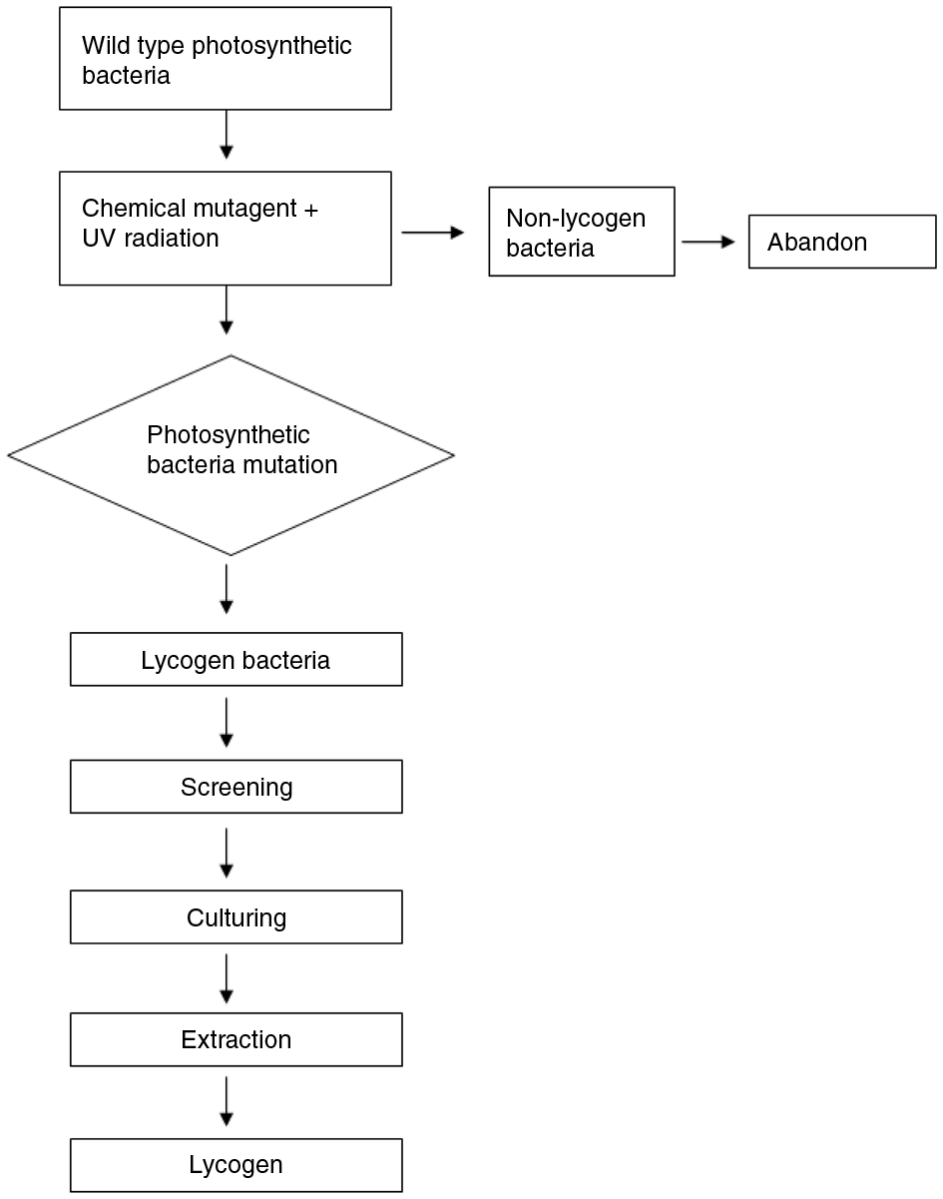
Flow chart of Lycogen production.

A mutant strain capable of producing colored carotenoids, in contrast to the colorless phenotype of the wild type, was isolated from among hundreds of strains using a chemical mutagen sodium azide (NaN_3_) (18) (Fig. 1). The isolated mutant was identified as *R. sphaeroides* (WL-APD911) and the sequence of its crtC was obtained and compared with that of the wild-type allele (Bioresource Collection and Research Center, Hsinchu, Taiwan).

### Sample preparation

The strain was grown at 30°C on Luria–Bertani (LB) agar plates. After harvesting, the bacterial broth was centrifuged and washed with ethanol. The bacterial residue was extracted in the dark with methanol at room temperature and then centrifuged at 7,500 rpm for 5 min. The supernatant was filtered through a filter paper and a 0.2-µm filter into a round flask. The color of the end supernatant was dark red. The methanol extract was filtered and the solvents removed under reduced pressure in a rotary evaporator to yield dried crude total extracts. The end crystalloid was stored at room temperature and preserved in darkness. The *R. sphaeroides* extract was named Lycogen™. The Lycogen™ powders were dissolved in CDCl_3_ for NMR analysis.

### NMR spectroscopy

All samples were dissolved in CDCl_3_ and sealed in 5-mm NMR tubes. TMS (tetramethylsilane) was used as the chemical shift reference for both ^1^H and ^13^C spectra. The NMR spectra were acquired on a Varian Inova Unity 500-MHz spectrometer with a double resonance probe. Approximately 5% D_2_O was used in a field-frequency lock to maintain long-term stability of 0.1 Hz for the static magnetic field. The ^1^H 90° pulse length was 8 µs and the ^13^C 90° pulse length was 10 µs. The spectral width was 11 ppm for the ^1^H spectra and 250 ppm for the ^13^C spectra. The acquisition times were 2 s and 1 s for the ^1^H and ^13^C spectra, respectively. Thirty-two scans and 2,000 scans were used for the 1D ^1^H and ^13^C spectra, respectively. For the 2D ^1^H–^1^H COSY spectra, the spectral widths for both dimensions were the same as that of the 1D spectrum, and the original data matrix was 256×2,048 with 32 scans for each FID. All experiments were conducted at room temperature. The collection time domain signals (FIDs) were subsequently Fourier transformed with Lorentzian line broadening factors of 1 and 2 Hz for the ^1^H and ^13^C spectra, respectively. For the ^1^H–^1^H COSY spectra, the data matrices were extended to 2,048×2,048 before Fourier transformation. The spectra were processed with the software built into the spectrometer.

ACD software was used to calculate ^1^H and ^13^C chemical shifts of the individual carotenoids, and the calculated chemical shifts and J couplings of each compound were used to assign the peaks in the experimental spectrum. The simulated spectra were then added up with a weighting coefficient for each spectrum, and the best fit was achieved using two different methods (least square and χ^2^).

### Primary skin irritation test with hamsters

The hamsters (*n*=3) were shaved on the trunk and lateral areas. Lycogen™ powder was resuspended in tetrahydrofuran (THF) to 20 µM and applied to intact skin under 1.7-cm diameter gauze patches. After 24, 48, and 72 h, skin response was scored for erythema and edema for each hamster. In addition, Lycogen™ was tested for its cumulative irritation. Lycogen™ was mixed with a gel containing Carbopo 1940, Tween 20, and neutralizing agents in TEA (Triethanolamine) buffer and applied to the skin for 30 days.

### DPPH (1,1-Diphenyl-2-picryl-hydrazyl) radical-scavenging activity

Free-radical scavenging activity was determined using the method described by Braca et al. (22). A freshly prepared DPPH solution, lycopene, and Lycogen™ at various concentrations (5, 10, 25, 50, and 100 µM) were mixed and incubated at 37°C for 30 min. The absorbance at 517 nm was determined, and the percentage of inhibitory activity was calculated as [(A_0_−A_1_)/A_0_]×100%, where A_0_ is the absorbance of control and A_1_ is the absorbance of the extract/standard. Finally, inhibition curves were constructed, and IC50 values were obtained.

## Results and discussion

The recent findings of the health-related properties of carotenoids and the increasing demand for natural products have spurred an intensive interest in the biotechnological overproduction of carotenoids in plants. Nevertheless, the carotenoid production through chemical synthesis or extraction from plants is limited by low yields that result in high production costs. This led to the study of the microbial production of carotenoids as an alternative that has shown better yields than other aforementioned methods. In our studies, we altered the crtC gene of *R. sphaeroides* by chemical mutagenesis and studied the difference between the wild-type and mutant strains.

**Fig. 2:**
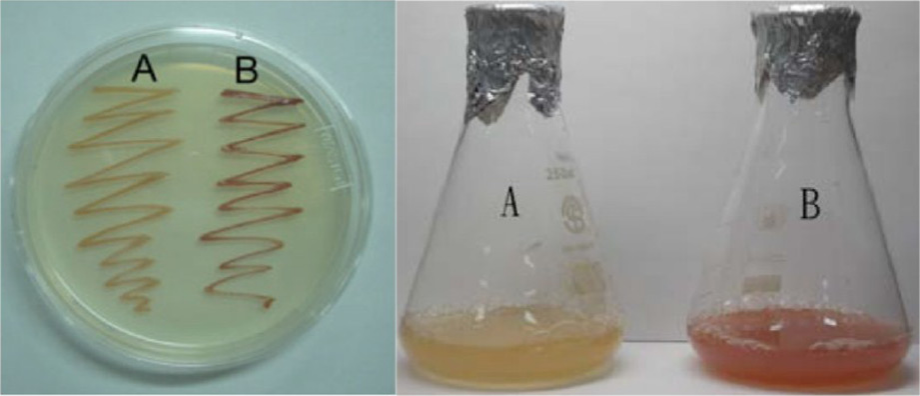
Culture of *R. sphaeroides* wild type (labeled as A) and WL-APD911 (labeled as B) in agar and broth medium.

**Fig. 3:**
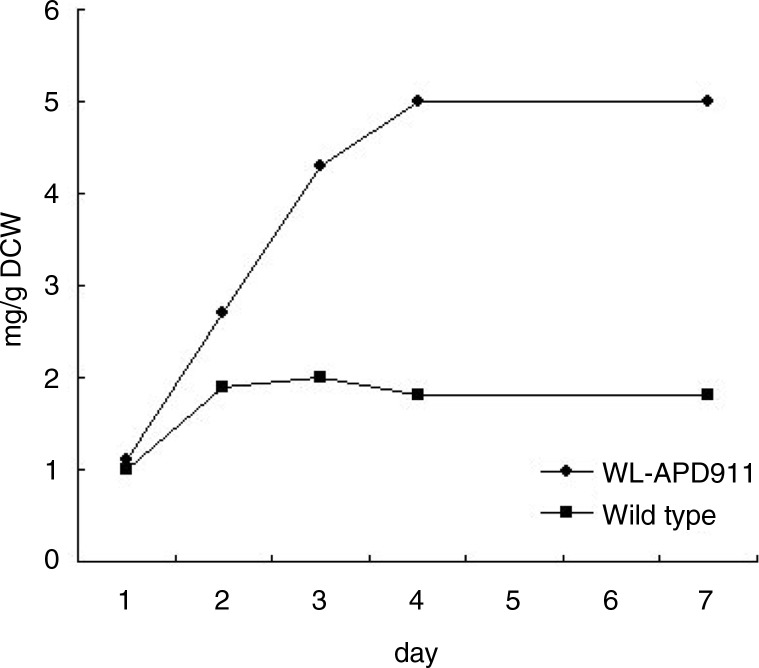
Carotenoid content in *R. sphaeroides* wild type and WL-APD911. The differences in the extracted carotenoid contents of the wild-type and mutant strains are shown. The mutant strain, WL-APD-911, displays a 3.5-fold increase in carotenoid content after a 4-day culture period, relative to the wild type. DCW, dry cell weight.

**Fig. 4:**
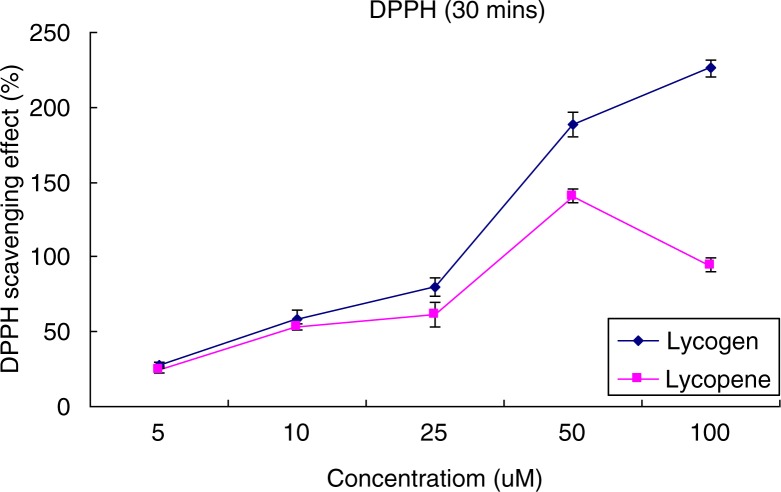
DPPH antioxidant test: Lycogen™ compared with lycopene.

Figure 2 shows the culture medium and agar plate of wild-type and mutant (WL-APD911) strains of *R. sphaeroides*. Based on visual inspection, we can easily observe the differences between these two strains with respect to the color compounds contained within them. Figure 3 shows the differences in the extracted carotenoid content between the wild-type and mutant strains. After a 4-day culture period, the mutant strain (WL-APD-911) displayed a 3.5-fold increase in carotenoid content, relative to the wild type. In the DPPH test, Lycogen™ showed more potent antioxidative activity than lycopene, particularly at higher concentrations (Fig. 4). In brief, the mutant strain yielded not only higher amounts of carotenoids but also highly potent carotenoids compared with the wild-type strain.

**Fig. 5:**
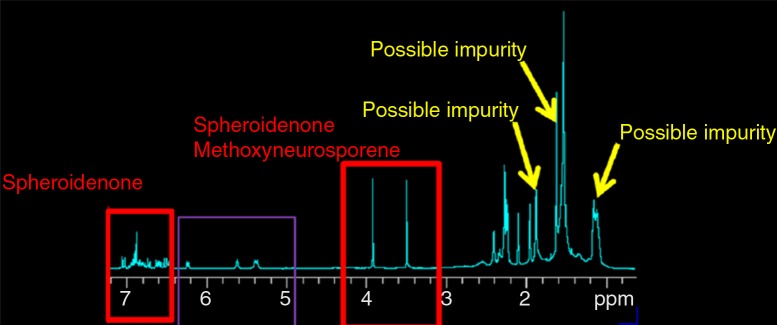
^1^H spectrum of the sample with assignment to compounds and possible impurities.

**Fig. 6:**
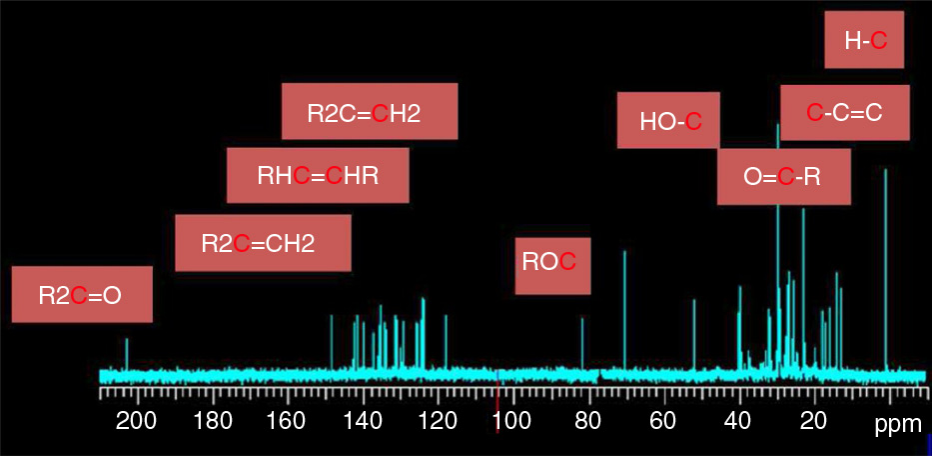
^13^C spectrum of the sample with assignment to different groups of carbon.

**Fig. 7:**
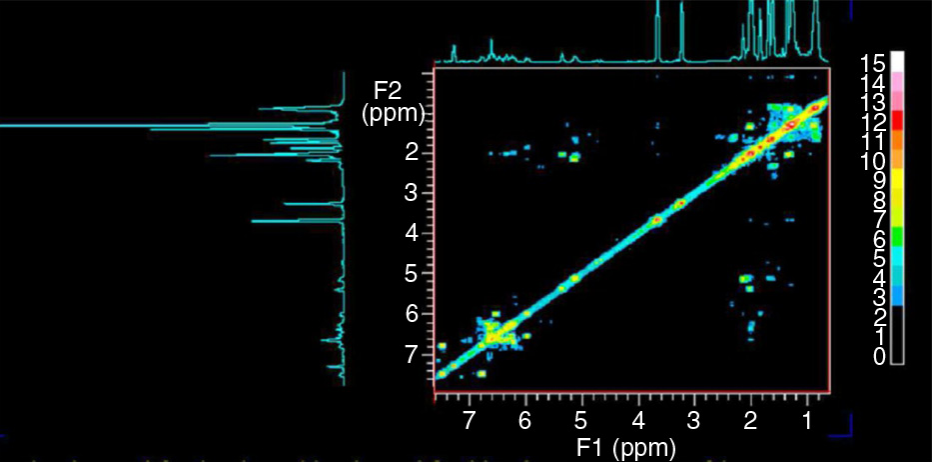
^1^H COSY spectrum of the sample. The fact that the peaks at 1.13, 1.62, and 1.83 ppm have no cross peaks with any other peak indicates that they represent impurities.

In addition to ξ-carotene and neurosporene, which have been reported by Wu and Liu (18), we found two other carotenoid derivatives in Lycogen™. The ^1^H and ^13^C chemical shifts of the two compounds are listed in Supplementary tables 1 and 2, respectively. The ^1^H, ^13^C 1D spectra and^1^H COSY spectrum and their assignments are given in Figs. 5–7). The components of the sample can be identified by comparing the ^1^H and ^13^C spectra with the standard spectra of the pure compounds as well as by fitting the simulated spectra with ACD software.

The ^1^H peak at 3.35 ppm represents methoxyneurosporene, and the peak at 3.89 ppm represents spheroidenone. In addition, the peaks at 1.13, 1.62, and 1.83 ppm represented none of the carotenoids and were attributed to impurities that could not be identified. In addition, from the known NMR spectral databases of *R. sphaeroides*, we were unable to assign carotenoids to these chemical shifts. Finally, the ^13^C peak at 203.4 ppm represented spheroidenone, the 5th carbon R2C=O.

**Fig. 8:**
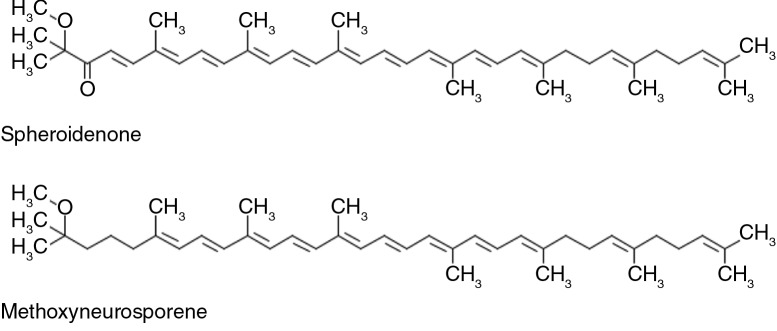
Structure of spheroidenone and methoxyneurosporene.

The above results were also consistent with ^1^H COSY. The absence of the cross peaks at 1.13, 1.62, and 1.83 ppm in the ^1^H COSY spectrum confirmed that those peaks were indeed the result of impurities. We therefore concluded that our sample contained approximately equal percentages of these two compounds. Quantitative analysis of these identified compounds based on their spectral intensities indicated that spheroidenone and methoxyneurosporene (Fig. 8) were major components (approximately 1:1); minimal quantities of other derivatives were also present in the sample.

**Fig. 9:**
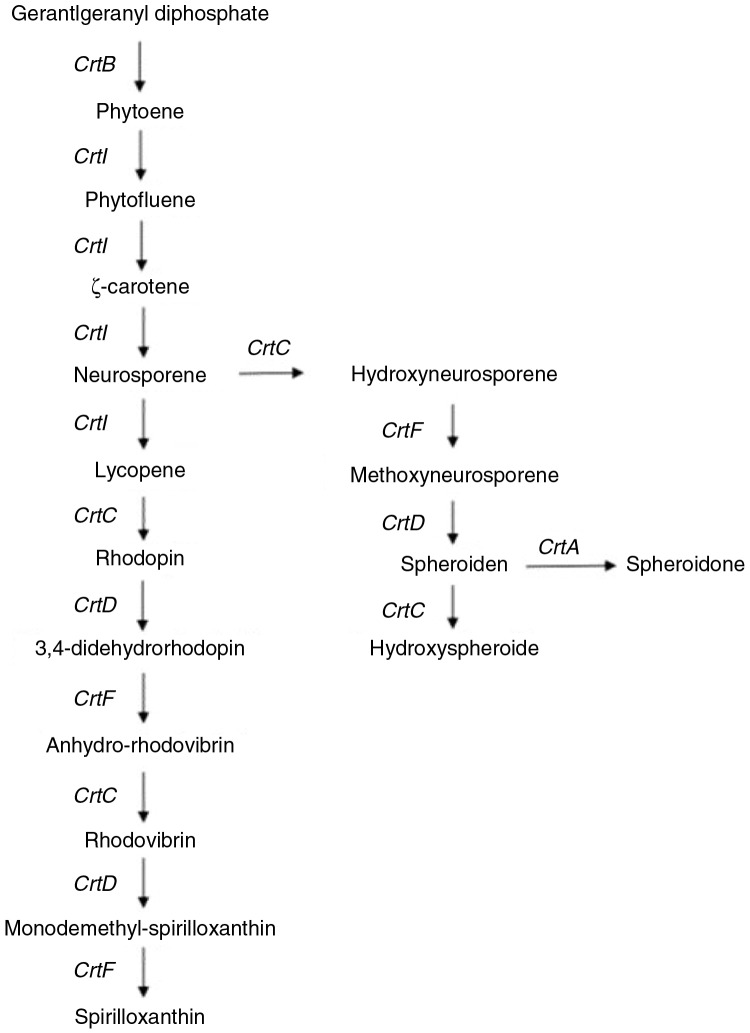
Synthetic pathway of Lycogen™.

Following several previous studies (23–26), this study used phototrophic bacteria as a carotenoid source. The mutant strain *R. sphaeroides* (WL-APD911) whose crtC gene has been mutated by random chemical mutagenesis provides a source of two carotenoid derivatives, methoxyneurosporene and spheroidenone (the pathway shown in Fig. 9). In *R. sphaeroides*, phytoene desaturase (CrtI) plays an important role in the synthesis of carotenoids (27–29); CrtI catalyses three desaturations to produce neurosporene, which is further modified by the CrtC/D/F/A enzymes in the spheroidene pathway (18).

Spheroidenone is one of the major types of carotenoids accumulating in variable amounts by *R. sphaeroides* (16), and methoxyneurosporene is a substance found in spheroidene biosynthesis (30). However, no previous studies have focused on the biological functions of these two compounds in animals.

Two other carotenoid derivatives contained in Lycogen™, ζ-carotene and neurosporene, have been reported in our previous studies (31). ζ-Carotene is the precursor of neurosporene, and neurosporene, in turn, is the precursor of lycopene (28, 32). The anti-inflammatory ability that has been attributed to Lycogen™ may result from the effects of lycopene; however, we did not observe any chemical shifts caused by lycopene in the NMR spectra of Lycogen™. We therefore suggest that other carotenoid derivatives also contribute to the LPS-induced anti-inflammatory properties previously observed on RAW 264.7 cells (31) and in DSS-induced colitis (21). In this study, we confirmed that spheroidenone and methoxyneurosporene were major components of Lycogen™. Therefore, methoxyneurosporene and spheroidenone may contribute to the biotechnological potential of Lycogen™, including antioxidation, anti-inflammation, the inhibition of melanogenesis, and the prevention of cisplatin-induced renal injury.

In the human organism, carotenoids are part of the antioxidant defense system. They interact synergistically with other antioxidants. Lycogen™, a mixture with major components including spheroidenone and methoxyneurosporene, presented with more potent antioxidative activity and less cytotoxicity (19) than single compound lycopene. This finding is compatible with a previous concept: Mixtures of carotenoids are more effective than a single compound (33). Thus, mixtures of carotenoids have the potential to be used as a carotenoid source.

**Fig. 10:**
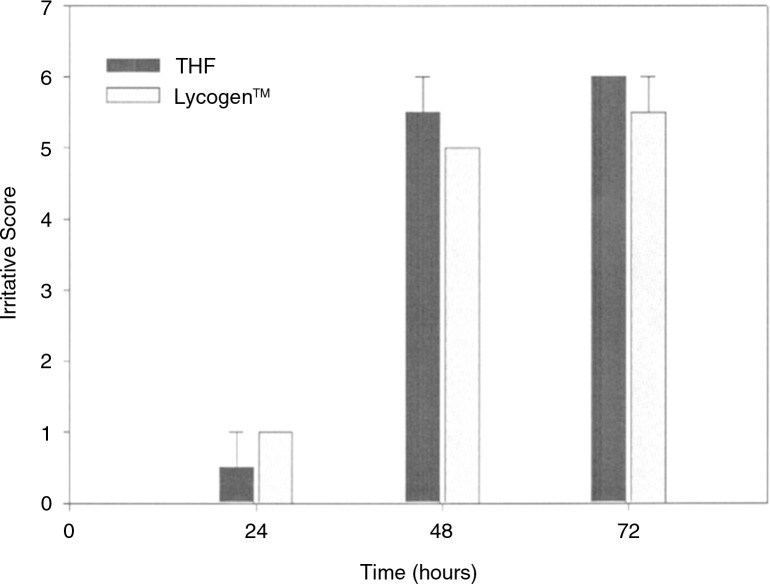
Irritative score of cumulative skin irritation at 24, 48, and 72 h in hamsters treated with THF and Lycogen™.

The biocompatibility of Lycogen™ was determined by the assessment of primary skin and a cumulative irritation test in hamster skins. Figure 10 shows the irritation responses of hamster skins to 20-mg Lycogen™ and tetrahydrofuran (THF as the solvent control) after 24, 48, and 72 h of treatment. THF caused irritative injury to hamster skins. In contrast, the administration of 20 mg Lycogen™ in THF led to no significant aggravation of skin irritation in hamsters (Table 1). Thus, treatment with 20-mg Lycogen™ should not lead to skin irritation in animals. Table 1 shows the assessment of cumulative skin irritation with Lycogen™ treatment. We found that there was no irritation response in hamster skins after 30 days of treatment with 0.2% Lycogen™, demonstrating that Lycogen™ has good biocompatibility. Thus, Lycogen™ can serve as a good candidate for a biological source of carotenoids, and it can be used as a natural material applicable in the cosmetic and pharmaceutical industries.

**Table 1:** Assessment of cumulative skin irritation after three days or 30 days with treatment of Lycogen™ in hamsters.

Treatment	Irritation score (Mean±SD)	Degree of irritation
Saline (3-day testing)	0.00±0.00	No irritation
THF (3-day testing)	4.00±0.47	Medium
20 mg Lycogen™ (3-day testing)	3.83±0.24	Medium
Gel without Lycogen™ (30-day testing)	0.00±0.00	No irritation
Gel with 0.2% Lycogen™ (30-day testing)	0.00±0.00	No irritation

## Conclusion

Lycogen™, an extract of *R. sphaeroides* (WL-APD911) whose crtC gene has been altered by chemical mutagenesis, has potent antioxidative and anti-inflammatory properties, as revealed in our previous studies. In this study, we confirmed the major carotenoid compounds contained in Lycogen™, including spheroidenone and methoxyneurosporene by NMR spectroscopy analysis. Methoxyneurosporene and spheroidenone may contribute to the biotechnological potential of Lycogen™, including antioxidation, anti-inflammation, the inhibition of melanogenesis, and the prevention of cisplatin-induced renal injury. In addition, the carotenoid content of this mutant strain of *R. sphaeroides* was 3.5-fold higher than that in normal strain. Furthermore, Lycogen™ from the mutant strain is more potent than lycopene from the wild-type strain. According to the assessment of primary skin and cumulative irritation tests of Lycogen™, Lycogen™ does not cause irritation in hamster skins. These findings suggest that this mutant strain has the potential to be used as an enriched carotenoid source.

## Supplementary Material

Extract from a mutant *Rhodobacter sphaeroides* as an enriched carotenoid source
