# Supplementary material for: Extract from a mutant Rhodobacter sphaeroides as an enriched carotenoid source
Source: Food Nutr Res. 2016 Mar 31;60:10.3402/fnr.v60.29580. doi: 10.3402/fnr.v60.29580 (PMC4818355; doi:10.3402/fnr.v60.29580)
Supplement: Extract from a mutant Rhodobacter sphaeroides as an enriched carotenoid source [file FNR-60-29580-s001.docx]

**Supplementary Table 1**. ^1^H chemical shift of methoxyneurosporene and spheroidenone.

| Methoxyneurosporene | | Spheroidenone | |
| --- | --- | --- | --- |
| Atom | Shift | Atom | Shift |
| 6 | 2.12 | 1 | 1.35 |
| 6 | 2.12 | 1 | 1.35 |
| 7 | 2.12 | 1 | 1.35 |
| 7 | 2.12 | 4 | 3.22 |
| 10 | 1.62 | 4 | 3.22 |
| 10 | 1.62 | 4 | 3.22 |
| 10 | 1.62 | 27 | 2.21 |
| 11 | 1.67 | 27 | 2.21 |
| 11 | 1.67 | 28 | 2.14 |
| 11 | 1.67 | 28 | 2.14 |
| 12 | 1.83 | 31 | 2.03 |
| 12 | 1.83 | 31 | 2.03 |
| 12 | 1.83 | 32 | 2.02 |
| 30 | 1.82 | 32 | 2.02 |
| 30 | 1.82 | 35 | 1.62 |
| 30 | 1.82 | 35 | 1.62 |
| 31 | 2.12 | 35 | 1.62 |
| 31 | 2.12 | 36 | 1.7 |
| 32 | 2.12 | 36 | 1.7 |
| 32 | 2.12 | 36 | 1.7 |
| 35 | 1.63 | 37 | 1.64 |
| 35 | 1.63 | 37 | 1.64 |
| 35 | 1.63 | 37 | 1.64 |
| 36 | 1.67 | 38 | 1.84 |
| 36 | 1.67 | 38 | 1.84 |
| 36 | 1.67 | 38 | 1.84 |
| 37 | 1.96 | 39 | 1.94 |
| 37 | 1.96 | 39 | 1.94 |
| 37 | 1.96 | 39 | 1.94 |
| 38 | 1.97 | 40 | 1.98 |
| 38 | 1.97 | 40 | 1.98 |
| 38 | 1.97 | 40 | 1.98 |
| 39 | 1.97 | 41 | 2 |
| 39 | 1.97 | 41 | 2 |
| 39 | 1.97 | 41 | 2 |
| 40 | 1.96 | 42 | 1.99 |
| 40 | 1.96 | 42 | 1.99 |
| 40 | 1.96 | 42 | 1.99 |
| 41 | 6.25 | 43 | 1.35 |
| 42 | 6.5 | 43 | 1.35 |
| 43 | 5.96 | 43 | 1.35 |
| 44 | 5.13 | 44 | 6.77 |
| 45 | 6.19 | 45 | 7.47 |
| 46 | 6.64 | 46 | 6.6 |
| 47 | 6.35 | 47 | 6.61 |
| 48 | 6.24 | 48 | 6.55 |
| 49 | 6.63 | 49 | 6.35 |
| 50 | 6.63 | 50 | 6.64 |
| 51 | 6.24 | 51 | 6.45 |
| 52 | 6.35 | 52 | 6.25 |
| 53 | 6.64 | 53 | 6.63 |
| 54 | 6.19 | 54 | 6.63 |
| 55 | 6.25 | 55 | 6.25 |
| 56 | 6.5 | 56 | 6.22 |
| 57 | 5.95 | 57 | 6.51 |
| 58 | 5.14 | 58 | 5.95 |
|  |  | 59 | 5.13 |
|  |  | 60 | 5.12 |

**Supplementary Table 2.**^13^C chemical shift of methoxyneurosporene and spheroidenone.

| Methoxyneurosporene | | Spheroidenone | |
| --- | --- | --- | --- |
| Atom | Shift | Atom | Shift |
| 26.08 | 3.77 | 1 | 26.08 |
| 2 | 80.18 | 2 | 80.18 |
| 4 | 53.04 | 4 | 53.04 |
| 5 | 203.3 | 5 | 203.3 |
| 7 | 126.54 | 7 | 126.54 |
| 8 | 146.94 | 8 | 146.94 |
| 9 | 133.91 | 9 | 133.91 |
| 10 | 140.75 | 10 | 140.75 |
| 11 | 124.46 | 11 | 124.46 |
| 12 | 140.77 | 12 | 140.77 |
| 13 | 135.99 | 13 | 135.99 |
| 14 | 136.17 | 14 | 136.17 |
| 15 | 130.64 | 15 | 130.64 |
| 16 | 139.13 | 16 | 139.13 |
| 17 | 136.69 | 17 | 136.69 |
| 18 | 132.61 | 18 | 132.61 |
| 19 | 129.49 | 19 | 129.49 |
| 20 | 130.12 | 20 | 130.12 |
| 21 | 131.56 | 21 | 131.56 |
| 22 | 135.97 | 22 | 135.97 |
| 23 | 135.08 | 23 | 135.08 |
| 24 | 124.92 | 24 | 124.92 |
| 25 | 126.49 | 25 | 126.49 |
| 26 | 139.58 | 26 | 139.58 |
| 27 | 32.97 | 27 | 32.97 |
| 28 | 27.4 | 28 | 27.4 |
| 29 | 125 | 29 | 125 |
| 30 | 135.25 | 30 | 135.25 |
| 31 | 31.94 | 31 | 31.94 |
| 32 | 26.55 | 32 | 26.55 |
| 33 | 123.88 | 33 | 123.88 |
| 34 | 131.77 | 34 | 131.77 |
| 35 | 17.68 | 35 | 17.68 |
| 36 | 25.67 | 36 | 25.67 |
| 37 | 23.41 | 37 | 23.41 |
| 38 | 24.13 | 38 | 24.13 |
| 39 | 12.75 | 39 | 12.75 |
| 40 | 12.91 | 40 | 12.91 |
| 41 | 12.97 | 41 | 12.97 |
| 42 | 12.85 | 42 | 12.85 |
| 43 | 26.08 | 43 | 26.08 |
